# Location, number and factors associated with cerebral microbleeds in an Italian-British cohort of CADASIL patients

**DOI:** 10.1371/journal.pone.0190878

**Published:** 2018-01-25

**Authors:** Serena Nannucci, Valentina Rinnoci, Giovanni Pracucci, Andrew D. MacKinnon, Francesca Pescini, Poneh Adib-Samii, Silvia Bianchi, Maria Teresa Dotti, Antonio Federico, Domenico Inzitari, Hugh S. Markus, Leonardo Pantoni

**Affiliations:** 1 NEUROFARBA Department, University of Florence, Florence, Italy; 2 Atkinson Morley Regional Neuroscience Centre, St George's University Hospitals NHS Foundation Trust, London, United Kingdom; 3 Stroke Unit, Azienda Ospedaliero Universitaria Careggi, Florence, Italy; 4 Clinical Neurosciences, St George's University of London, London, United Kingdom; 5 Department of Medical, Surgical and Neurological Sciences, University of Siena, Siena, Italy; 6 Institute of Neuroscience, Italian National Research Council, Florence, Italy; 7 Stroke Research Group, Department of Clinical Neurosciences, University of Cambridge, Cambridge, United Kingdom; 8 'L. Sacco' Department of Biomedical and Clinical Sciences, University of Milan, Milan, Italy; Taipei Veterans General Hospital, TAIWAN

## Abstract

**Background and purpose:**

The frequency, clinical correlates, and risk factors of cerebral microbleeds (CMB) in Cerebral Autosomal Dominant Arteriopathy with Subcortical Infarcts and Leukoencephalopathy (CADASIL) are still poorly known. We aimed at determining the location and number of CMB and their relationship with clinical manifestations, vascular risk factors, drugs, and other neuroimaging features in CADASIL patients.

**Methods:**

We collected clinical data by means of a structured proforma and centrally evaluated CMB on magnetic resonance gradient echo sequences applying the Microbleed Anatomical Rating Scale in CADASIL patients seen in 2 referral centers in Italy and United Kingdom.

**Results:**

We evaluated 125 patients. CMB were present in 34% of patients and their presence was strongly influenced by the age. Twenty-nine percent of the patients had CMB in deep subcortical location, 22% in a lobar location, and 18% in infratentorial regions. After adjustment for age, factors significantly associated with a higher total number of CMB were hemorrhagic stroke, dementia, urge incontinence, and statins use (this latter not confirmed by multivariate analysis). Infratentorial and deep CMB were associated with dementia and urge incontinence, lobar CMB with hemorrhagic stroke, dementia, and statins use. Unexpectedly, patients with migraine, with or without aura, had a lower total, deep, and lobar number of CMB than patients without migraine.

**Discussion:**

CMB formation in CADASIL seems to increase with age. History of hemorrhagic stroke, dementia, urge incontinence, and statins use are associated with a higher number of CMB. However, these findings need to be confirmed by longitudinal studies.

## Introduction

Cerebral Autosomal Dominant Arteriopathy with Subcortical Infarcts and Leukoencephalopathy (CADASIL; MIM 125310) is an inherited cerebral microangiopathy caused by *NOTCH3* mutations [1]. The disease is characterized by migraine, frequently with aura, stroke, psychiatric and cognitive disturbances, usually with a progressive course leading to disability and dementia [2]. Usually, strokes are ischemic and of the lacunar type. However, CADASIL patients with intracerebral hemorrhage have also been described [3]. From the neuroimaging point of view, the most frequent features are a severe leukoencephalopathy involving the anterior temporal poles and external capsules and lacunar infarcts [4–8]. Cerebral microbleeds (CMB) on gradient echo MRI sequences have been reported in CADASIL [9–11], but their frequency, clinical correlates, and risk factors remain poorly defined.

The aims of this study were to investigate the location and number of CMB in CADASIL using an internationally proposed scale, the Microbleed Anatomical Rating Scale (MARS) [12], and to explore factors associated with their presence.

## Methods

CADASIL patients seen in two referral centers for cerebrovascular diseases (both comprising a stroke unit and a clinic dedicated to patients with sporadic and genetic small vessel diseases) in Florence, Italy, and London, United Kingdom, were included. All patients had a typical *NOTCH3* mutation which resulted in a change in a cysteine amino acid in one of the extra-cellular epidermal growth factor-like repeats, apart from one patient with a family history who had granular osmiophilic material (GOM) on electron microscopy examination of a skin biopsy. For all patients, information on clinical features, vascular risk factors, and use of antithrombotic drugs and statins was collected prospectively by means of a structured proforma. For this study, we reviewed each available brain MRI scan performed on 1.5 Tesla magnet for clinical purposes at the time of the clinical assessment. For each patient, MRI included at least axial and/or sagittal T1-weighted images, axial gradient echo T2*-weighted images, axial FLuid Attenuated Inversion Recovery (FLAIR) images, and axial or coronal T2-weighted fast spin-echo images. The study was approved by the local ethics committees (Comitato Etico Azienda Ospedaliero Universitaria Careggi and English Multicentre Research Ethics Committee) and all patients gave a written informed consent for genetic testing and participation in the study. No minor was enrolled in the study.

### Definition of clinical variables

Stroke and TIA were defined according to current criteria [13,14]. Psychiatric disorders were recorded as present in the case of any of the following: 1) diagnosis of a psychiatric disease by a certified specialist (psychiatrist, geriatrician, or neurologist); 2) previous or current use of antipsychotic or antidepressant drugs or psychotherapy (the sole use of anxiolytics was not sufficient); 3) mood or behavior disorders referred by the patient or his/her family, not immediately related to bereavement, and that had interfered for at least 6 months with daily or work activities. Cognitive disorders were recorded as present if one of the following instances was present: 1) previous diagnosis of mild cognitive impairment or dementia by a certified specialist (geriatrician or neurologist); 2) overt cognitive impairment emerged during the first evaluation at our centers; 3) presence of cognitive decline from a previously normal status referred by a next of kin or by the patient and confirmed by neuropsychological testing. Neuropsychological cognitive performances were judged impaired if the patient scored 1.5 standard deviations below the age- and education-corrected means in at least one cognitive test. Headache and migraine with and without aura were defined according to the Headache Classification Committee of the International Headache Society [15]. Seizures were defined according to the International League Against Epilepsy Commission Report [16]. Acute reversible encephalopathy was defined as an episode of altered consciousness associated with seizures and/or hallucinations after exclusion of infectious, metabolic and paraneoplastic causes [17]. The presence of hypertension was ascertained based on a previous diagnosis or according to the World Health Organization Guidelines [18] as a systolic blood pressure ≥140 mmHg and/or a diastolic blood pressure ≥90 mmHg on multiple blood pressure measurements, taken on several separate occasions. Diabetes mellitus was defined according to the American Diabetes Association criteria [19]. Hypercholesterolemia included total cholesterol >200, low-density lipoprotein >130, and high-density lipoprotein <35 mg/dl (each value had to be found elevated in at least two measurements) [20]. Hyperhomocysteinemia was defined when elevated values were found according to local laboratory norms. Smoking was considered as present in case of current or previous history. Urge incontinence was clinically defined by each center investigators.

### Definition of neuroimaging variables

MRI scans were reviewed by a single trained observer (SN). CMB were defined as small, round, well-defined lesions, hypointense and associated with blooming effect on MR gradient echo T2* sequences, 2–10 mm in diameter [12,21]. They were further evaluated by applying the MARS [12]. This scale classifies CMB into infratentorial (including brainstem and cerebellum), deep (basal ganglia, thalamus, external and internal capsules, corpus callosum, deep and periventricular white matter), and lobar (frontal, parietal, temporal, occipital, and insula, including cortical and subcortical regions with U fibers). According to the MARS, we classified CMB in definite, as previously defined, and possible, when the above mentioned characteristics were less clear [12]; only definite CMB were considered in our analyses. Presence and severity of white matter changes on FLAIR or T2-weighted images were evaluated according to a modified Fazekas scale [22]: grade 1: single lesions <10 mm; areas of ‘grouped’ lesions <20 mm in any diameter; grade 2: single lesions between 10 and 20 mm; areas of ‘grouped’ lesions more than 20 mm in any diameter; no more than ‘connecting bridges’ between individual lesions; grade 3: single lesions or confluent areas of hyperintensity >20 mm in any diameter. The presence of hyperintense lesions on FLAIR and T2-weighted images in the anterior temporal lobe white matter, external capsule, and pons was also recorded. Lacunar infarcts were defined as focal hyperintensities on T2-weighted images, 3–15 mm in diameter, and with a corresponding hypointensity on T1-weighted images [21].

### Statistical analysis

Intra-rater agreement for number and location of CMB was determined. Twenty-eight anonymized scans were reviewed by the same observer 6 months after the first evaluation blinded to clinical information. Agreement was calculated using the intraclass correlation coefficient. Reliability was high for each MARS category: 0.888 for total CMB, 0.904 for infratentorial, 0.943 for deep, and 0.810 for lobar CMB. Descriptive analyses were used to characterize the baseline sample in terms of demographic, clinical and neuroimaging features. Analysis of variance (ANOVA) or Pearson correlation were applied to test for associations of clinical and neuroimaging variables with the number and location of CMB. Analyses were adjusted for age using UNIANOVA (IBM, SPSS Statistics version 24). Significantly associated variables were included in a stepwise linear regression to identify independent predictors of number and location of CMB. Because of the small number of CMB in each site specified in the MARS, we grouped them in the 3 location categories defined in the scale (deep subcortical, lobar, infratentorial).

## Results

One hundred and twenty-five CADASIL patients (91 probands and 34 relatives) were included in the analysis. Most patients had *NOTCH3* mutations on exon 4, followed by exons 11, 3, and 20. Demographic, clinical and neuroimaging features are summarized in Table 1. The mean (SD) age at assessment was 50.6 (14.2) years and 56 patients (45%) were males. The mean (SD) age at onset of the disease was 33.4 (17.1) years. The most frequent symptom at onset was migraine with or without aura, followed by stroke and psychiatric disturbances. Eight patients (3 probands and 5 relatives) were asymptomatic at the time of assessment having had presymptomatic genetic testing. All of these patients had the MRI abnormalities typical of CADASIL: 1 proband and 2 relatives had leukoencephalopathy involving the temporal pole and the external capsule, while 2 probands and 3 relatives had leukoencephalopathy extended only to the temporal pole.

**Table 1:** Demographic, clinical and neuroimaging characteristics of the 125 CADASIL patients enrolled in the study.

**Age at assessment, years (mean ± SD)**	**50.6 ± 14.2**
**Age at onset, years (mean ± SD)**	**33.4 ± 17.1**
**Age at first stroke, years (mean ± SD)**	**49.7 ± 12.8**
**Male gender, n (%)**	**56/125 (45)**
**Ischemic stroke, n (%)**	**49/125 (39)**
**TIA, n (%)**	**19/125 (15)**
**Recurrent ischemic events, n (%)**	**30/125 (24)**
**Hemorrhagic stroke, n (%)**	**3/125 (2)**
**Cognitive impairment, n (%)**	**46/125 (37)**
**Dementia, n (%)**	**9/123 (7)**
**Psychiatric disturbances, n (%)**	**54/125 (43)**
**Migraine, n (%)**	**84/125 (67)**
**Migraine with aura, n (%)**	**60/125 (48)**
**Prolonged aura, n (%)**	**16/120 (13)**
**Seizures, n (%)**	**11/125 (9)**
**Encephalopathy, n (%)**	**15/125 (12)**
**Urge incontinence, n (%)**	**28/116 (24)**
**Hypertension, n (%)**	**37/125 (30)**
**Diabetes, n (%)**	**7/125 (6)**
**Hypercholesterolemia, n (%)**	**70/121 (58)**
**History of smoking, n (%)**	**59/124 (48)**
**Hyperhomocysteinemia, n (%)**	**26/99 (26)**
**Antithrombotic treatment, n (%)**	**83/119 (70)**
**Statins use, n (%)**	**49/122 (40)**
**Fazekas grade 1, n (%)**	**22/125 (18)**
**Fazekas grade 2, n (%)**	**18/125 (14)**
**Fazekas grade 3, n (%)**	**82/125 (66)**
**Pontine leukoaraiosis, n (%)**	**65/122 (53)**
**Temporal pole involvement, n (%)**	**110/125 (88)**
**External capsule involvement, n (%)**	**96/125 (77)**
**Lacunar infarcts, n (%)**	**80/125 (64)**

CADASIL: Cerebral Autosomal Dominant Arteriopathy with Subcortical Infarcts and Leukoencephalopathy; TIA: transient ischemic attack

**Fig 1:**
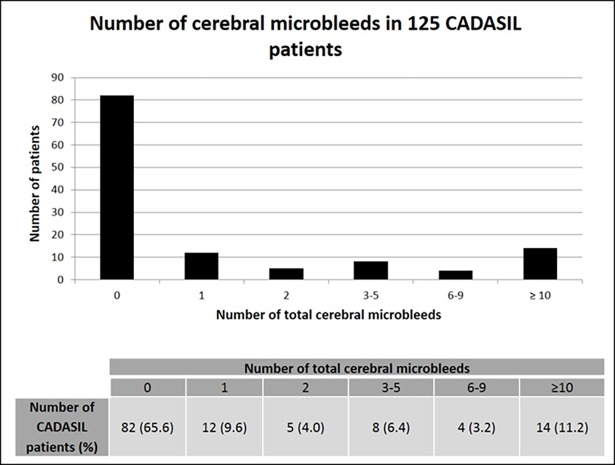
Total number of cerebral microbleeds in 125 CADASIL patients. Proportions of patients with different numbers of CMB are reported in the bar graph and in the table.

**Fig 2:**
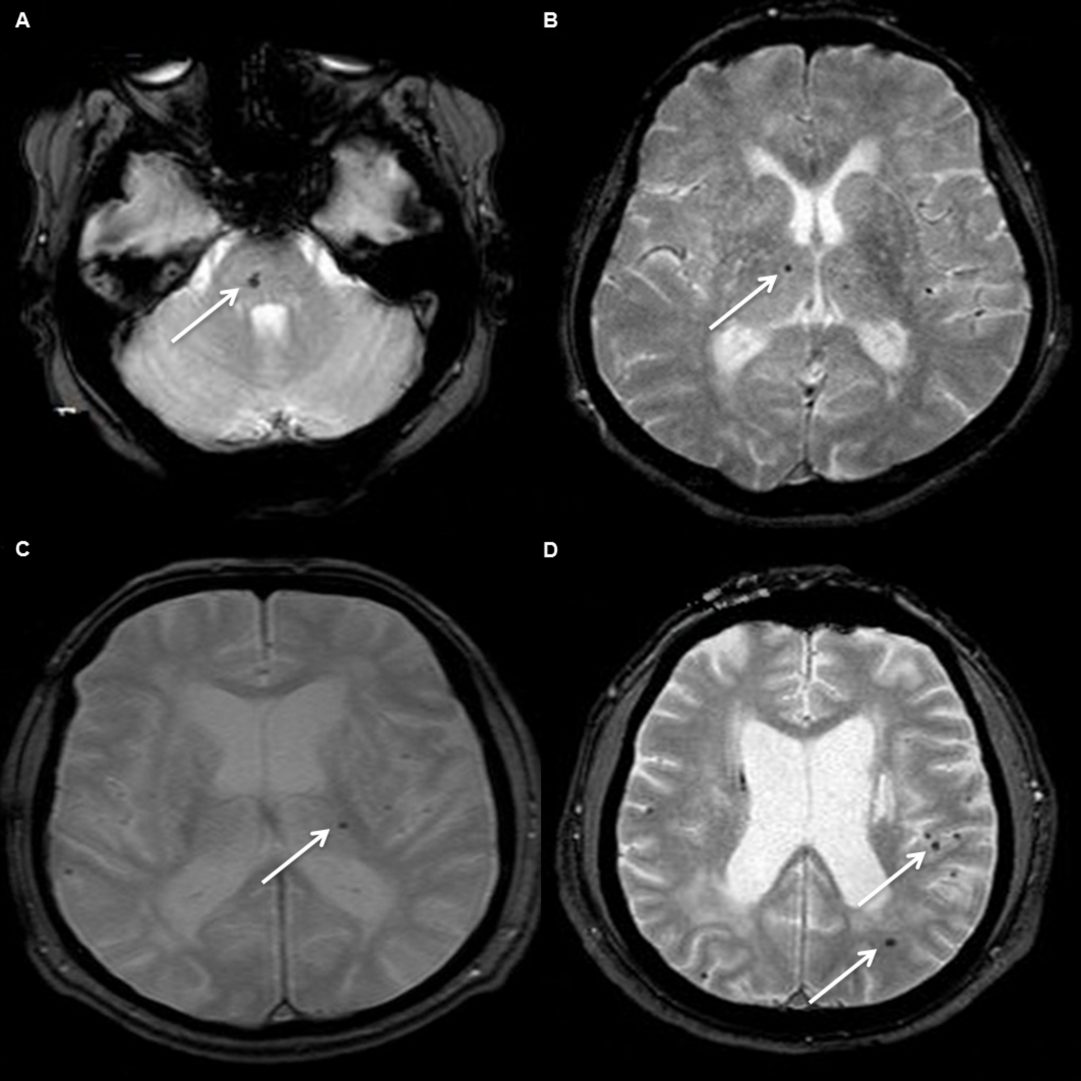
Examples of different locations of cerebral microbleeds in our sample of CADASIL patients. T2*-weighted magnetic resonance images showing cerebral microbleeds (white arrows) in infratentorial (A), deep subcortical (B, C) and lobar locations (D).

Forty-three patients (34%) had at least one CMB (range 1–73) (Fig 1). Twenty-nine percent had CMB in a deep subcortical location (range 1–24), most frequently in the thalamus, 22% had CMB in a lobar location (1–37), mainly temporal, and 18% in an infratentorial location (1–12) (Fig 2). The associations of number of total, infratentorial, deep, and lobar CMB with demographic, clinical and imaging variables are shown in Tables 2 and 3.

**Table 2:** Correlation between the total number of cerebral microbleeds and demographics, drugs, clinical and other neuroimaging features in 125 CADASIL patients.

	Number of CMB mean (SD)	p*	p**
**Male gender** (yes vs no)	3.8 (9.9) vs 3.8 (10.9)	0.981	0.676
**Ischemic stroke** (yes vs no)	5.5 (11.3) vs 2.7 (9.7)	0.144	0.980
**TIA** (yes vs no)	7.8 (17.1) vs 3.0 (8.6)	0.065	0.662
**Recurrent ischemic events** (yes vs no)	5.6 (9.8) vs 3.2 (10.6)	0.286	0.501
**Hemorrhagic stroke** (yes vs no)	25.3 (21.8) vs 3.3 (9.5)	**<0.001**	**0.001**
**Cognitive impairment** (yes vs no)	8.8 (15.3) vs 0.8 (3.6)	**<0.001**	0.140
**Dementia** (yes vs no)	22.4 (24.2) vs 2.4 (6.9)	**<0.001**	**<0.001**
**Psychiatric disturbances** (yes vs no)	4.0 (8.9) vs 3.6 (11.5)	0.833	0.575
**Migraine** (yes vs no)	1.3 (4.4) vs 8.8 (16.0)	**<0.001**	**<0.001**
**Migraine with aura** (yes vs no)	1.1 (4.7) vs 6.3 (13.3)	**0.005**	**0.004**
**Prolonged aura** (yes vs no)	0.4 (1.0) vs 4.1 (10.9)	0.169	0.141
**Seizures** (yes vs no)	9.6 (15.0) vs 3.2 (9.7)	0.051	0.265
**Acute reversible encephalopathy** (yes vs no)	1.2 (2.8) vs 4.1 (11.0)	0.306	0.246
**Urge incontinence** (yes vs no)	12.0 (17.8) vs 1.4 (5.2)	**<0.001**	**0.008**
**Hypertension** (yes vs no)	8.0 (15.7) vs 2.0 (6.4)	**0.003**	0.650
**Diabetes** (yes vs no)	7.3 (15.8) vs 3.6 (10.0)	0.363	0.870
**Hypercholesterolemia** (yes vs no)	5.0 (10.0) vs 2.5 (11.2)	0.199	0.721
**History of smoking** (yes vs no)	3.4 (9.1) vs 4.2 (11.6)	0.680	0.821
**Hyperhomocysteinemia** (yes vs no)	6.4 (15.8) vs 3.4 (8.9)	0.236	0.386
**Antithrombotic treatment** (yes vs no)	5.0 (12.0) vs 1.6 (6.1)	0.111	0.450
**Statins use** (yes vs no)	3.8 (7.5) vs 3.9 (12.2)	0.967	**0.009**
**Fazekas grade 1** (yes vs no)	0.0 (0.0) vs 4.6 (11.3)	**0.036**	0.429
**Fazekas grade 2** (yes vs no)	0.2 (0.5) vs 4.4 (11.3)		
**Fazekas grade 3** (yes vs no)	5.7 (12.4) vs 0.1 (0.3)		
**Pontine leukoaraiosis** (yes vs no)	5.9 (13.0) vs 0.9 (3.9)	**0.007**	0.918
**Temporal pole involvement** (yes vs no)	4.0 (10.7) vs 2.5 (8.2)	0.619	0.968
**External capsule involvement** (yes vs no)	4.9 (11.6) vs 0.0 (0.2)	**0.026**	0.106
**Lacunar infarcts** (yes vs no)	5.8 (12.5) vs 0.2 (1.2)	**0.003**	0.916

CADASIL: Cerebral Autosomal Dominant Arteriopathy with Subcortical Infarcts and Leukoencephalopathy; CMB: cerebral microbleeds; SD: standard deviations; TIA: transient ischemic attack

* ANOVA

** ANOVA adjusted for age

p values reported in bold are statistically significant

**Table 3:** Correlation between the number of infratentorial, deep, and lobar cerebral microbleeds and drugs, clinical and other neuroimaging features in 125 CADASIL patients.

	Infratentorial		Deep		Lobar	
	Number of CMB mean (SD)	p*	Number of CMB mean (SD)	p*	Number of CMB mean (SD)	p*
**Male gender** (yes vs no)	0.4 (1.1) vs 0.6 (1.9)	0.486	1.3 (2.8) vs 1.9 (4.8)	0.578	2.1 (6.8) vs 1.2 (4.8)	0.193
**Ischemic stroke** (yes vs no)	0.7 (1.6) vs 0.4 (1.6)	0.915	2.0 (3.9) vs 1.4 (4.1)	0.482	2.7 (7.3) vs 0.9 (4.5)	0.687
**TIA** (yes vs no)	1.3 (2.8) vs 0.4 (1.3)	0.261	3.5 (6.3) vs 1.3 (3.4)	0.375	3.0 (8.7) vs 1.4 (5.1)	0.873
**Recurrent ischemic events** (yes vs no)	0.9 (1.7) vs 0.4 (1.6)	0.857	2.8 (4.9) vs 1.3 (3.7)	0.869	1.9 (4.8) vs 1.5 (6.1)	0.182
**Hemorrhagic stroke** (yes vs no)	1.7 (2.9) vs 0.5 (1.6)	0.534	6.7 (6.1) vs 1.5 (3.9)	0.127	17.0 (17.1) vs 1.3 (4.9)	**<0.001**
**Cognitive impairment** (yes vs no)	1.2 (2.3) vs 0.1 (0.7)	0.105	3.7 (5.7) vs 0.4 (1.9)	0.073	3.9 (9.0) vs 0.3 (1.5)	0.374
**Dementia** (yes vs no)	3.2 (3.8) vs 0.3 (1.1)	**<0.001**	8.8 (8.2) vs 1.1 (3.0)	**<0.001**	10.4 (13.6) vs 1.0 (4.1)	**<0.001**
**Psychiatric disturbances** (yes vs no)	0.6 (1.4) vs 0.5 (1.7)	0.859	1.9 (4.0) vs 1.4 (4.1)	0.978	1.5 (5.2) vs 1.7 (6.2)	0.346
**Migraine** (yes vs no)	0.3 (1.1) vs 0.9 (2.2)	0.125	0.8 (2.7) vs 3.3 (5.6)	**0.001**	0.2 (0.9) vs 4.5 (9.5)	**<0.001**
**Migraine with aura** (yes vs no)	0.3 (1.1) vs 0.8 (1.9)	0.106	0.6 (2.8) vs 2.5 (4.8)	**0.007**	0.2 (0.9) vs 3.0 (7.8)	**0.008**
**Prolonged aura** (yes vs no)	0.2 (0.7) vs 0.5 (1.6)	0.409	0.1 (0.3) vs 1.7 (4.0)	0.084	0.1 (0.2) vs 1.9 (6.3)	0.236
**Seizures** (yes vs no)	0.7 (1.5) vs 0.5 (1.6)	0.709	3.4 (5.0) vs 1.4 (3.9)	0.498	5.4 (10.7) vs 1.3 (5.0)	0.113
**Acute reversible encephalopathy** (yes vs no)	0.3 (0.6) vs 0.6 (1.7)	0.472	0.8 (2.1) vs 1.7 (4.2)	0.346	0.1 (0.3) vs 1.8 (6.1)	0.242
**Urge incontinence** (yes vs no)	1.6 (2.8) vs 0.2 (0.8)	**0.011**	5.0 (6.8) vs 0.7 (2.1)	**0.003**	5.3 (10.2) vs 0.6 (3.1)	0.062
**Hypertension** (yes vs no)	1.0 (2.3) vs 0.3 (1.1)	0.909	3.1 (5.6) vs 1.0 (3.1)	0.910	3.9 (9.2) vs 0.7 (3.2)	0.497
**Diabetes** (yes vs no)	0.0 (0.0) vs 0.5 (1.6)	0.057	2.0 (2.8) vs 1.6 (4.1)	0.360	5.3 (13.1) vs 1.4 (5.1)	0.384
**Hypercholesterolemia** (yes vs no)	0.7 (1.6) vs 0.3 (1.7)	0.973	2.3 (4.3) vs 0.8 (3.6)	0.699	2.0 (5.8) vs 1.3 (6.0)	0.383
**History of smoking** (yes vs no)	0.4 (1.4) vs 0.6 (1.8)	0.518	1.4 (3.7) vs 1.9 (4.4)	0.593	1.6 (5.5) vs 1.7 (6.1)	0.878
**Hyperhomocysteinemia** (yes vs no)	1.1 (2.6) vs 0.4 (1.3)	0.164	2.6 (5.7) vs 1.5 (3.8)	0.422	2.7 (7.7) vs 1.5 (5.4)	0.571
**Antithrombotic treatment** (yes vs no)	0.7 (1.9) vs 0.2 (0.9)	0.721	2.1 (4.5) vs 0.7 (2.9)	0.547	2.2 (6.9) vs 0.7 (2.6)	0.425
**Statins use** (yes vs no)	0.7 (1.5) vs 0.4 (1.7)	0.375	2.1 (4.3) vs 1.3 (3.9)	0.211	1.0 (2.3) vs 2.1 (7.3)	**0.001**
**Fazekas grade 1** (yes vs no)	0.0 (0.0) vs 0.6 (1.7)	0.731	0.0 (0.0) vs 1.9 (4.4)	0.529	0.0 (0.0) vs 2.0 (6.3)	0.427
**Fazekas grade 2** (yes vs no)	0.0 (0.0) vs 0.6 (1.7)		0.1 (0.2) vs 1.9 (4.3)		0.1 (0.3) vs 1.9 (6.2)	
**Fazekas grade 3** (yes vs no)	0.8 (1.9) vs 0.0 (0.0)		2.5 (4.8) vs 0.0 (0.2)		2.5 (7.0) vs 0.0 (0.2)	
**Pontine leukoaraiosis** (yes vs no)	0.7 (1.8) vs 0.1 (0.7)	0.984	2.4 (4.6) vs 0.4 (2.1)	0.771	2.7 (7.8) vs 0.3 (1.2)	0.998
**Temporal pole involvement** (yes vs no)	0.5 (1.6) vs 0.3 (1.3)	0.886	1.7 (4.2) vs 0.9 (3.1)	0.785	1.7 (6.0) vs 1.3 (3.8)	0.876
**External capsule involvement** (yes vs no)	0.7 (1.8) vs 0.0 (0.0)	0.400	2.1 (4.5) vs 0.0 (0.0)	0.218	2.1 (6.5) vs 0.0 (0.2)	0.083
**Lacunar infarcts** (yes vs no)	0.8 (1.9) vs 0.0 (0.0)	0.597	2.4 (4.8) vs 0.2 (1.2)	0.988	2.6 (7.1) vs 0.0 (0.0)	0.747

CADASIL: Cerebral Autosomal Dominant Arteriopathy with Subcortical Infarcts and Leukoencephalopathy; CMB: cerebral microbleeds; SD: standard deviations; TIA: transient ischemic attack

* ANOVA adjusted for age

p values reported in bold are statistically significant

Among patients on antithrombotic treatment, only one was receiving anticoagulant treatment. Three unrelated patients (39, 54, and 67 years) experienced hemorrhagic strokes: 2 patients had a thalamo-capsular hemorrhage and one of these had also a hemispheric cerebellar hemorrhage, while the third patient suffered from an interpeduncular cistern subarachnoid hemorrhage. None of them was receiving antiplatelets, anticoagulants or statins at the time of hemorrhage, but all were affected by hypertension. CMB were present in all of these patients both in lobar and deep subcortical locations.

For each location, the presence and number of CMB was strongly associated with age (Pearson correlation, p<0.001). Regarding the total number of CMB, Pearson correlation coefficients were the following: 0.478 for age at time of assessment, 0.450 for age at disease onset, and 0.318 for age at first stroke. Therefore, in Tables 2 and 3 age-adjusted p values are presented. After adjustment for age, a history of hemorrhagic stroke, dementia, urge incontinence, and statins use were factors significantly associated with a higher total number of CMB (Table 2). Considering location, infratentorial and deep CMB were significantly associated with dementia and urge incontinence, while lobar CMB with hemorrhagic stroke, dementia, and statins use (Table 3). Patients with migraine, with or without aura, had significantly fewer total CMB than patients who did not suffer from migraine (1.4 **±** 4.5 versus 8.5 **±** 15.9, p<0.001) (Table 2). This negative association was found also considering separately deep and lobar locations and remained statistically significant after correction for age (Tables 2 and 3). On the multivariate analysis, each variable that was statistically significant at the univariate analysis maintained an independent predictive value with the exception of the statins use. No correlation between other neuroimaging characteristics typical of CADASIL and number and location of CMB was found after adjustment for age in our cohort.

## Discussion

In this binational series of CADASIL patients, we found that one third of patients had CMB and that these ones occurred throughout subcortical, infratentorial and lobar regions. We also found that history of hemorrhagic stroke, dementia, and urge incontinence were independently associated with a higher number of CMB, while patients with migraine had a lower burden of CMB in comparison with non-migraineurs. Regarding many aspects, our study confirmed the results obtained in previous studies focused on CMB in CADASIL. In particular, the prevalence of CMB in this CADASIL cohort is in keeping with other CADASIL series [9,11] that reported prevalence ranging between 25% and 69% [7,10]. In spite of the absence of a clearly predominant location of CMB in our sample, the most frequent one was thalamic, followed by the lobar one, mainly temporal, as also described in other series [7,9,10,23]. Finally, the number of CMB was strongly positively correlated with age in our patients. A strict correlation between CMB and increasing age has been widely described in the literature, both in sporadic cerebral microangiopathy [24–26] and in other CADASIL samples [7,9,10,23,27]. In CADASIL patients, one could speculate that this strong correlation of CMB with age might be at least partially explained by the disease progression and a higher prevalence of hypertension in more advanced ages.

In our study, dementia was associated with CMB in each analyzed location and this association was also confirmed after a multivariate analysis, in contrast with data reported by others [11]. An association between number of CMB and cognitive decline in specific domains, such as memory and executive function, was clearly reported by Liem and colleagues [28]. Of note, urge incontinence was more frequent in CADASIL patients with a higher number of CMB in infratentorial and deep locations, independently from the age. An association between urinary urgency and cerebral small vessel disease has been reported in the literature, but mainly related with severe white matter changes in patients with sporadic microangiopathy [29]. Little is known about a possible correlation between CMB and urinary disturbances in subjects with sporadic small vessel disease and no data are available about other CADASIL populations.

No correlation was found between the use of antithrombotic drugs and CMB after adjustment for age. On a first-step of analysis, we found that statins use was associated with a higher number of total and lobar CMB. In different populations, such as patients suffering from acute cerebrovascular events, an association between high-dose atorvastatin and a higher number of CMB was found [30]. However, in our sample, the association between statins use and CMB disappeared on a multivariate analysis.

A quite surprising result was the association between migraine, both with and without aura, and a lower number of CMB, particularly in deep and lobar location. To the best of our knowledge, this is the first study investigating the correlation between CMB and migraine. This finding is not of immediate interpretation from a pathogenic point of view and needs to be confirmed in other patient series.

We did not find a relation between CMB and other features of cerebral small vessel disease. At present, we do not have a precise explanation for this. The hypothesis could be that the pathophysiological mechanisms underlying CMB in CADASIL are partially different from those responsible for leukoaraiosis and lacunar infarct. However, we had no mean to test this hypothesis in this study.

The study has a number of strengths. First, it included a considerable number of patients taking into account that CADASIL is a rare disease. Second, the assessment of CMB was performed by applying a structured scale, allowing quantification and localization. Third, this is the first study to investigate the relationship of considerable number of clinical variables with CMB in CADASIL patients. In the literature, only data about cognitive profile and some psychiatric disturbances are reported [23,28,31,32], but data are missing about many other clinical features typical of CADASIL. Fourth, our sample is composed of patients from different countries and this could make our results more generalizable to the CADASIL patient community.

This study has also limitations. It is a cross-sectional study and therefore it is not possible to draw definitive conclusions about the real predictors of the location and number of CMB in CADASIL patients. Longitudinal studies are needed to confirm our observations. Another possible limitation is the low prevalence in our sample of some of the examined clinical manifestations that resulted associated with a higher number of CMB (in particular, only 3 patients had hemorrhagic stroke and only 9 patients had dementia).

In summary, our study shows that CMB are common in CADASIL and may have a number of clinical correlates. It is possible that their use may help in optimal planning of treatments such as anti-platelet agents but assessing this will require prospective longitudinal studies and randomized controlled trials.

## Supporting information

S1 File Dataset. (XLS)
